# The Physician’s Quarterly Record and Statement

**Published:** 1881-12

**Authors:** 


The Physicians' Quarterly Record and Statement. By Wm. Jefferson Guernsey, M. D. Price 80 cents; pp. 50. David Heston, publisher: Philadelphia (Station "F"), 1881.

Nearly every physician has his own method of keeping his accounts; some using a scrap of paper, others a large ledger. To facilitate easy and accurate keeping of doctor's books has been a problem which many have tried to solve; we think the "Record," arranged by Dr. Guernsey, about the best we have yet seen, and can recommend it to the profession. Its chief merit, in our eyes, is that it does away with the necessity of looking over old accounts, in making up bills. The bill and the account are on same page, and when bill is torn off, you know the account has been rendered; when the "statement" is torn off, you know the account has been paid. It is a very complete "record."

Pamphlets received.—We return thanks for the following:

"PRIVATE CARE OF THE INSANE." By Ralph L. Parsons, N. Y.

"SEWERS AND SEWER GAS." By D. H. Beckwith, Cleveland, Ohio.

INTRODUCTORY LECTURE, at Pulte Med. College. By Prof. Wm. Owens, Cincinnati.

THE ADDRESS OF THE PRESIDENT OF THE HOM. INTERNATIONAL CONVENTION, held in London, July, 1881. By Prof. R. Hughes.

